# 3-Carbamothioylpyridinium iodide

**DOI:** 10.1107/S1600536809035892

**Published:** 2009-09-09

**Authors:** Shahzad Sharif, Mehmet Akkurt, Islam Ullah Khan, Shafqat Nadeem, Syed Ahmed Tirmizi, Saeed Ahmad

**Affiliations:** a Materials Chemistry Laboratory, Department of Chemistry, Government College University, Lahore 54000, Pakistan; b Department of Physics, Faculty of Arts and Sciences, Erciyes University, 38039 Kayseri, Turkey; c Department of Chemistry, Quaid-i-azam University, Islamabad, Pakistan; d Department of Chemistry, University of Engineering and Technology, Lahore 54890, Pakistan

## Abstract

In the crystal of the title salt, C_6_H_7_N_2_S^+^·I^−^, inversion-related cations form an *R*_2_^2^(8) dimer linked by a pair of N—H⋯S hydrogen bonds. Pairs of iodide anions are located between adjacent cation dimers and are linked to them by way of N—H⋯I hydrogen bonds. This results in zigzag chains propagating in [001] lying parallel to the *bc* plane.

## Related literature

For graph-set theory, see: Bernstein *et al.* (1995 ▶). 
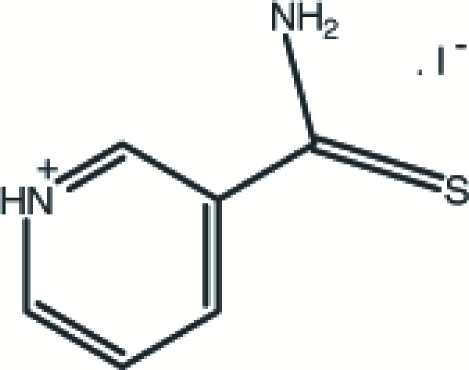


## Experimental

### 

#### Crystal data

C_6_H_7_N_2_S^+^·I^−^
*M* *_r_* = 266.11
Triclinic, 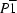

*a* = 4.4024 (3) Å
*b* = 8.1943 (5) Å
*c* = 12.6815 (8) Å
α = 102.485 (2)°
β = 96.496 (2)°
γ = 102.288 (2)°
*V* = 430.31 (5) Å^3^
*Z* = 2
Mo *K*α radiation
μ = 3.89 mm^−1^
*T* = 296 K
0.17 × 0.15 × 0.14 mm

#### Data collection

Bruker Kappa APEXII CCD diffractometer
Absorption correction: none
8839 measured reflections
2087 independent reflections
1890 reflections with *I* > 2σ(*I*)
*R*_int_ = 0.020

#### Refinement

*R*[*F* ^2^ > 2σ(*F* ^2^)] = 0.019
*wR*(*F* ^2^) = 0.047
*S* = 1.04
2087 reflections
91 parameters
H-atom parameters constrained
Δρ_max_ = 0.65 e Å^−3^
Δρ_min_ = −0.43 e Å^−3^

### 

Data collection: *APEX2* (Bruker, 2007 ▶); cell refinement: *SAINT* (Bruker, 2007 ▶); data reduction: *SAINT*; program(s) used to solve structure: *SIR97* (Altomare *et al.*, 1999 ▶); program(s) used to refine structure: *SHELXL97* (Sheldrick, 2008 ▶); molecular graphics: *ORTEP-3 for Windows* (Farrugia, 1997 ▶); software used to prepare material for publication: *WinGX* (Farrugia, 1999 ▶) and *PLATON* (Spek, 2009 ▶).

## Supplementary Material

Crystal structure: contains datablocks global, I. DOI: 10.1107/S1600536809035892/hb5088sup1.cif

Structure factors: contains datablocks I. DOI: 10.1107/S1600536809035892/hb5088Isup2.hkl

Additional supplementary materials:  crystallographic information; 3D view; checkCIF report

## Figures and Tables

**Table 1:** Hydrogen-bond geometry (Å, °)

*D*—H⋯*A*	*D*—H	H⋯*A*	*D*⋯*A*	*D*—H⋯*A*
N1—H*N*1⋯I1^i^	0.86	2.62	3.444 (2)	161
N2—H2*A*⋯I1^ii^	0.86	3.04	3.747 (3)	140
N2—H2*B*⋯S1^iii^	0.86	2.58	3.420 (3)	164

Symmetry codes: (i) 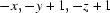; (ii) 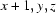; (iii) 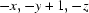.

**Fig. 1.**
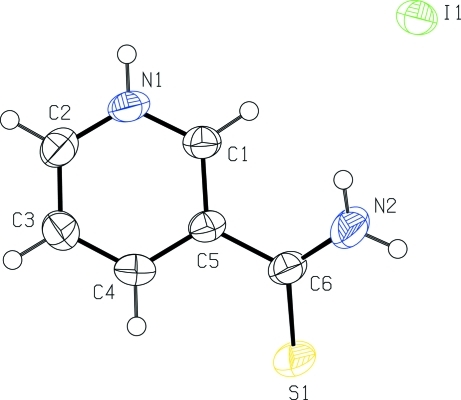
The molecular structure of (I) with displacement ellipsoids for the non-H atoms drawn at the 50% probability level.

**Fig. 2.**
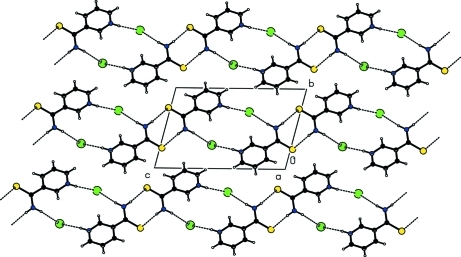
Packing diagram for (I) viewed down the a axis, showing the R22(8) dimer motif further linked by N—H···I hydrogen bonds between the adjacent dimers thorough the iodide anions to form an infinite chain in the [010] direction. Hydrogen atoms not involved in the showed interactions have been omitted for clarity.

**Fig. 3.**
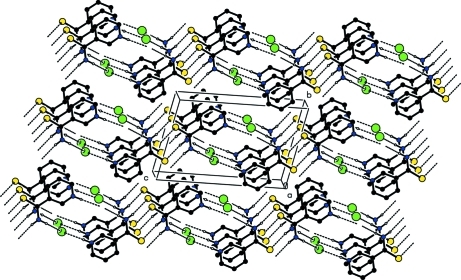
A view of the packing and hydrogen bonding of (I). Hydrogen atoms not involved in the showed interactions have been omitted for clarity.

## supplementary crystallographic information

### Comment

In the present study we attempted to prepare a palladium(II) iodide complex with thionicotinamide, but it is surprising to note that the resulting compound is a simple salt of pyridine. Here we report the crystal structure of the salt (I).

In the title compund (I), (Fig. 1), the bond lengths and angles are entirely as expected. In the crystal structure of (I), two crystallographically independent cations form a dimer through N—H···S hydrogen bonds. The two iodide anions are located between two adjacent dimers and forms N—H···I hydrogen bonds with two iodide anions from each dimer. Thus, the molecules linked in the form of zigzag in the layers parallel to the *bc* plane along the *b* axis (Fig. 2 and Fig. 3, Table 1).

### Experimental

The title compound was prepared by adding 2 equivalents of thionicotinamide in 15 ml methanol to a solution of K_2_[PdCl_4_] (0.326 g) in 15 ml of water followed by addition of 2 equivalents of potassium iodide in water after half an hour stirring. The dark brown solution was the stirred for one hour. The resulting solution was filtrated and filtrate was kept at room temperature for crystallization. The brown product obtained from water-methanol mixture wasre-dissolved in methanol, which on slow evaporation yielded light brown crystals of (I).

### Refinement

All H atoms were located geometrically and treated as riding with C—H = 0.93 Å and N—H = 0.86 Å with *U*_iso_(H) = 1.2*U*_eq_(C, N).

### Crystal data

**Table:**

C_6_H_7_N_2_S^+^·I^−^	*Z* = 2
*M**_r_* = 266.11	*F*(000) = 252
Triclinic, *P*1	*D*_x_ = 2.054 Mg m^−^^3^
Hall symbol: -P 1	Mo *K*α radiation, λ = 0.71073 Å
*a* = 4.4024 (3) Å	Cell parameters from 6584 reflections
*b* = 8.1943 (5) Å	θ = 2.6–28.3°
*c* = 12.6815 (8) Å	µ = 3.89 mm^−^^1^
α = 102.485 (2)°	*T* = 296 K
β = 96.496 (2)°	Irregular chunk, light brown
γ = 102.288 (2)°	0.17 × 0.15 × 0.14 mm
*V* = 430.31 (5) Å^3^	

### Data collection

**Table:**

Bruker Kappa APEXII CCD diffractometer	1890 reflections with *I* > 2σ(*I*)
Radiation source: sealed tube	*R*_int_ = 0.020
graphite	θ_max_ = 28.3°, θ_min_ = 2.6°
phi and ω scans	*h* = −5→5
8839 measured reflections	*k* = −9→10
2087 independent reflections	*l* = −16→16

### Refinement

**Table:**

Refinement on *F*^2^	Primary atom site location: structure-invariant direct methods
Least-squares matrix: full	Secondary atom site location: difference Fourier map
*R*[*F*^2^ > 2σ(*F*^2^)] = 0.019	Hydrogen site location: inferred from neighbouring sites
*wR*(*F*^2^) = 0.047	H-atom parameters constrained
*S* = 1.04	*w* = 1/[σ^2^(*F*_o_^2^) + (0.0227*P*)^2^ + 0.219*P*] where *P* = (*F*_o_^2^ + 2*F*_c_^2^)/3
2087 reflections	(Δ/σ)_max_ = 0.001
91 parameters	Δρ_max_ = 0.65 e Å^−^^3^
0 restraints	Δρ_min_ = −0.42 e Å^−^^3^

### Special details

**Table:**

Geometry. Bond distances, angles *etc*. have been calculated using the rounded fractional coordinates. All su's are estimated from the variances of the (full) variance-covariance matrix. The cell e.s.d.'s are taken into account in the estimation of distances, angles and torsion angles
Refinement. Refinement on *F*^2^ for ALL reflections except those flagged by the user for potential systematic errors. Weighted *R*-factors *wR* and all goodnesses of fit *S* are based on *F*^2^, conventional *R*-factors *R* are based on *F*, with *F* set to zero for negative *F*^2^. The observed criterion of *F*^2^ > σ(*F*^2^) is used only for calculating -*R*-factor-obs *etc*. and is not relevant to the choice of reflections for refinement. *R*-factors based on *F*^2^ are statistically about twice as large as those based on *F*, and *R*-factors based on ALL data will be even larger.

### Fractional atomic coordinates and isotropic or equivalent isotropic displacement parameters (Å^2^)

**Table:**

	*x*	*y*	*z*	*U*_iso_*/*U*_eq_	
S1	0.1191 (2)	0.24949 (10)	−0.01595 (5)	0.0620 (3)	
N1	0.3984 (5)	0.2110 (3)	0.37108 (16)	0.0423 (6)	
N2	0.2489 (8)	0.5132 (3)	0.1550 (2)	0.0708 (12)	
C1	0.3138 (6)	0.3026 (3)	0.30244 (18)	0.0377 (7)	
C2	0.5245 (6)	0.0778 (3)	0.3413 (2)	0.0449 (8)	
C3	0.5737 (7)	0.0300 (3)	0.2358 (2)	0.0479 (8)	
C4	0.4852 (6)	0.1197 (3)	0.1621 (2)	0.0438 (8)	
C5	0.3541 (5)	0.2583 (3)	0.19426 (17)	0.0345 (6)	
C6	0.2468 (6)	0.3524 (3)	0.11482 (19)	0.0395 (7)	
I1	−0.08685 (4)	0.70787 (2)	0.39126 (1)	0.0426 (1)	
H1	0.22840	0.39550	0.32730	0.0450*	
HN1	0.36980	0.23970	0.43790	0.0510*	
H2	0.57890	0.01770	0.39200	0.0540*	
H2A	0.31010	0.55920	0.22390	0.0850*	
H2B	0.18910	0.57340	0.11260	0.0850*	
H3	0.66530	−0.06150	0.21410	0.0570*	
H4	0.51400	0.08680	0.08980	0.0530*	

### Atomic displacement parameters (Å^2^)

**Table:**

	*U*^11^	*U*^22^	*U*^33^	*U*^12^	*U*^13^	*U*^23^
S1	0.1014 (6)	0.0573 (4)	0.0263 (3)	0.0304 (4)	−0.0032 (3)	0.0045 (3)
N1	0.0534 (12)	0.0464 (12)	0.0256 (9)	0.0090 (10)	0.0078 (9)	0.0083 (9)
N2	0.131 (3)	0.0457 (14)	0.0331 (12)	0.0312 (15)	−0.0095 (14)	0.0070 (10)
C1	0.0430 (12)	0.0388 (12)	0.0296 (11)	0.0094 (10)	0.0060 (9)	0.0055 (9)
C2	0.0541 (15)	0.0409 (13)	0.0377 (13)	0.0076 (11)	0.0003 (11)	0.0132 (11)
C3	0.0572 (15)	0.0428 (14)	0.0441 (14)	0.0204 (12)	0.0045 (12)	0.0057 (11)
C4	0.0539 (14)	0.0471 (14)	0.0291 (11)	0.0151 (12)	0.0083 (10)	0.0033 (10)
C5	0.0377 (11)	0.0371 (11)	0.0261 (10)	0.0063 (9)	0.0030 (8)	0.0063 (9)
C6	0.0470 (13)	0.0426 (13)	0.0283 (11)	0.0095 (11)	0.0046 (9)	0.0098 (9)
I1	0.0421 (1)	0.0492 (1)	0.0351 (1)	0.0154 (1)	0.0065 (1)	0.0034 (1)

### Geometric parameters (Å, °)

**Table:**

S1—C6	1.661 (2)	C2—C3	1.366 (4)
N1—C1	1.337 (3)	C3—C4	1.379 (4)
N1—C2	1.330 (3)	C4—C5	1.386 (3)
N2—C6	1.304 (4)	C5—C6	1.489 (3)
N1—HN1	0.8600	C1—H1	0.9300
N2—H2B	0.8600	C2—H2	0.9300
N2—H2A	0.8600	C3—H3	0.9300
C1—C5	1.383 (3)	C4—H4	0.9300
			
I1···C1^i^	3.639 (3)	C1···C3^i^	3.433 (4)
I1···C2^ii^	3.818 (3)	C2···I1^x^	3.818 (3)
I1···N2	3.694 (3)	C2···I1^iv^	3.793 (3)
I1···N1^iii^	3.444 (2)	C3···C1^ix^	3.433 (4)
I1···C2^iv^	3.794 (3)	C4···S1^vii^	3.564 (3)
I1···H1	3.1600	C1···H2A	2.5200
I1···H2^v^	3.1900	H1···H2A	2.0800
I1···H2A^i^	3.0400	H1···I1	3.1600
I1···H2A	3.1100	H1···N2	2.5800
I1···H2^iv^	3.3800	HN1···I1^iii^	2.6200
I1···HN1^iii^	2.6200	H2···I1^xi^	3.1900
S1···N2^vi^	3.420 (3)	H2···I1^iv^	3.3800
S1···C4^vii^	3.564 (3)	H2A···H1	2.0800
S1···H3^viii^	3.0100	H2A···I1	3.1100
S1···H2B^vi^	2.5800	H2A···I1^ix^	3.0400
S1···H4	2.8000	H2A···C1	2.5200
N1···I1^iii^	3.444 (2)	H2B···S1^vi^	2.5800
N2···S1^vi^	3.420 (3)	H3···S1^viii^	3.0100
N2···I1	3.694 (3)	H4···S1	2.8000
N2···H1	2.5800	H4···H4^viii^	2.3800
C1···I1^ix^	3.639 (3)		
			
C1—N1—C2	123.4 (2)	C4—C5—C6	121.6 (2)
C1—N1—HN1	118.00	S1—C6—C5	119.92 (18)
C2—N1—HN1	118.00	N2—C6—C5	116.3 (2)
C6—N2—H2A	120.00	S1—C6—N2	123.7 (2)
H2A—N2—H2B	120.00	N1—C1—H1	120.00
C6—N2—H2B	120.00	C5—C1—H1	120.00
N1—C1—C5	119.6 (2)	N1—C2—H2	120.00
N1—C2—C3	119.5 (2)	C3—C2—H2	120.00
C2—C3—C4	118.9 (2)	C2—C3—H3	121.00
C3—C4—C5	121.0 (2)	C4—C3—H3	121.00
C1—C5—C4	117.7 (2)	C3—C4—H4	120.00
C1—C5—C6	120.6 (2)	C5—C4—H4	119.00
			
C2—N1—C1—C5	0.7 (4)	C3—C4—C5—C1	−0.4 (4)
C1—N1—C2—C3	0.3 (4)	C3—C4—C5—C6	−177.8 (2)
N1—C1—C5—C4	−0.6 (4)	C1—C5—C6—S1	−147.9 (2)
N1—C1—C5—C6	176.8 (2)	C1—C5—C6—N2	29.5 (4)
N1—C2—C3—C4	−1.2 (4)	C4—C5—C6—S1	29.4 (3)
C2—C3—C4—C5	1.3 (4)	C4—C5—C6—N2	−153.1 (3)

Symmetry codes: (i) *x*−1, *y*, *z*; (ii) *x*, *y*+1, *z*; (iii) −*x*, −*y*+1, −*z*+1; (iv) −*x*+1, −*y*+1, −*z*+1; (v) *x*−1, *y*+1, *z*; (vi) −*x*, −*y*+1, −*z*; (vii) −*x*, −*y*, −*z*; (viii) −*x*+1, −*y*, −*z*; (ix) *x*+1, *y*, *z*; (x) *x*, *y*−1, *z*; (xi) *x*+1, *y*−1, *z*.

### Hydrogen-bond geometry (Å, °)

**Table:**

*D*—H···*A*	*D*—H	H···*A*	*D*···*A*	*D*—H···*A*
N1—HN1···I1^iii^	0.86	2.62	3.444 (2)	161
N2—H2A···I1^ix^	0.86	3.04	3.747 (3)	140
N2—H2B···S1^vi^	0.86	2.58	3.420 (3)	164

Symmetry codes: (iii) −*x*, −*y*+1, −*z*+1; (ix) *x*+1, *y*, *z*; (vi) −*x*, −*y*+1, −*z*.
